# Recent advances in the role of toll-like receptors and TLR agonists in immunotherapy for human glioma

**DOI:** 10.1007/s13238-014-0112-6

**Published:** 2014-11-21

**Authors:** Shuanglin Deng, Shan Zhu, Yuan Qiao, Yong-Jun Liu, Wei Chen, Gang Zhao, Jingtao Chen

**Affiliations:** 1 Institute of Translational Medicine, the First Hospital, Jilin University, Changchun, 130031 China; 2 Department of Neurosurgery, the First Hospital, Jilin University, Changchun, 130031 China; 3 MedImmune, Gaithersburg, MD 20878 USA; 4 Department of Hematology-Oncology and BMT Department of Pediatrics, University of Minnesota Medical School, Minneapolis, MN 55455 USA

**Keywords:** glioma, toll-like receptor, agonist, central nervous system, immunotherapy

## Abstract

Gliomas are extremely aggressive brain tumors with a very poor prognosis. One of the more promising strategies for the treatment of human gliomas is targeted immunotherapy where antigens that are unique to the tumors are exploited to generate vaccines. The approach, however, is complicated by the fact that human gliomas escape immune surveillance by creating an immune suppressed microenvironment. In order to oppose the glioma imposed immune suppression, molecules and pathways involved in immune cell maturation, expansion, and migration are under intensive clinical investigation as adjuvant therapy. Toll-like receptors (TLRs) mediate many of these functions in immune cell types, and TLR agonists, thus, are currently primary candidate molecules to be used as important adjuvants in a variety of cancers. In animal models for glioma, TLR agonists have exhibited antitumor properties by facilitating antigen presentation and stimulating innate and adaptive immunity. In clinical trials, several TLR agonists have achieved survival benefit, and many more trials are recruiting or ongoing. However, a second complicating factor is that TLRs are also expressed on cancer cells where they can participate instead in a variety of tumor promoting activities including cell growth, proliferation, invasion, migration, and even stem cell maintenance. TLR agonists can, therefore, possibly play dual roles in tumor biology. Here, how TLRs and TLR agonists function in glioma biology and in anti-glioma therapies is summarized in an effort to provide a current picture of the sophisticated relationship of glioma with the immune system and the implications for immunotherapy.

## INTRODUCTION

Gliomas are the most common neoplasms occurring in the central nervous system (CNS), and are one of the most aggressive types of human cancer. Histological classification of gliomas according to the World Health Organization consists of grades I–IV (Louis et al., 2007), with grade I corresponding to a benign tumor and grades II to IV increasing in malignancy. Grade III and grade IV gliomas are considered malignant and have a very poor prognosis. Glioblastoma multiforme (GBM; grade IV), is the most aggressive form of human glioma, and the mean survival of these patients remains dismally at approximately 12 months despite intensive and comprehensive therapies (Louis et al., 2007).

One of the more promising therapeutic strategies for the treatment of human gliomas is immunotherapy. Elucidation of the molecular basis for immune response is, therefore, currently under rigorous analysis. Molecules fundamental to eliciting an immune response in various immune cell types are toll-like receptors (TLRs) and correspondingly, their receptor agonists. TLRs are an evolutionarily conserved family of pattern-recognition receptors with 10 functional members (TLR 1–10) in human that are expressed on a variety of immune cell types. Toll-like receptor agonists are pathogen-associated molecular patterns (PAMPs) that bind to TLRs with specificity and in a typical scenario, initiate an immune response. The complicating factor in glioma biology is that the same TLRs are also found to be expressed on glioma cells themselves. Further complicating the issue is the fact that specific family members, such as TLR2, TLR4, and TLR9, following detection of corresponding TLR agonists, have a tumor promoting role in the biology of these tumors.

Triggering TLRs to generate an immune response is therefore a primary goal in immunotherapy for cancer in general. The design of current strategies is to exploit antigens uniquely presented by a tumor type so that the vaccine will be specific, but to use TLR agonists as adjuvants to potentially enhance the response. TLR agonists have been well-described and furthermore have greater affinities for different TLRs so that they can be used to specifically probe immune response *in vivo*. Currently, agonists, such as BCG (TLR1, 2, 4, and 6), MPL (TLR4), poly(I:C) (TLR3), imiquimod (TLR7), and CpG (TLR9), have been tested and reported to initiate therapeutic effects to varying degrees in clinical trials for colon cancer (Vermorken et al., 1999), urothelial carcinoma (Sharma et al., 2008), non-small-cell lung cancer (Vansteenkiste et al., 2007), lymphoma (Brody et al., 2010), melanoma (Adams et al., 2008), and a number of other cancers.

Many TLR agonists have been introduced into animal models of glioma to investigate immunotherapies and their underlying mechanisms. Since the expression of TLRs on glioma cells has been associated with some tumor promoting activities, it is important to summarize the major findings concerning TLRs and TLR agonists in glioma models for a more comprehensive view of this field.

## TLRS AND TLR AGONISTS

The Toll gene was initially described in *Drosophila* and found to mediate dorsoventral embryonic development and innate immune functions (Anderson et al., 1985). The human homolog of Toll was subsequently cloned in 1997, and its role in the human adaptive immune response was determined (Medzhitov et al., 1997). Shortly thereafter, the first toll-like receptor, TLR4, was discovered (Poltorak et al., 1998). Currently, a total of 13 *TLR* genes have been identified in human and mouse genomes; TLR1–10 are functional in human, and TLR1–9 and TLR11–13 are functional in mouse.

Expression of the TLRs varies with immune cell type. Among human antigen presenting cells (APCs), TLR7, 9, and 10 are expressed on plasmacytoid dendritic cells (pDCs), whereas all TLRs, with the exception of TLR9, are expressed on myeloid derived DCs (mDCs). In human adaptive immune system, TLR1, 2, 3, 4, 5, 7, and 9 (Caron et al., 2005; Hornung et al., 2002; Zarember and Godowski, 2002) are expressed on T cells, and TLR5 and 8 (Crellin et al., 2005; Kabelitz, 2007) are expressed on regulatory T cells (Treg), a cell type critical to the maintenance of immune homeostasis. Finally, activated and memory B cells express significant levels of TLR1, 6, 7, 9, and 10 but low levels of TLR2 (Agrawal and Gupta, 2011; Bernasconi et al., 2003; Hornung et al., 2002; Mansson et al., 2006).

TLR agonists are generally microbial molecules that stimulate TLR receptors to initiate specific immunoactivity. The most frequently studied of these agonists include lipopolysaccharides (LPS; TLR4 agonist), lipopeptides (TLR1, TLR2, and TLR6 agonists), flagellin (TLR5 agonist), single stranded (TLR7 and TLR8 agonist) or double stranded (ds) RNA (TLR3 agonist), and DNA containing the CpG motif (TLR9 agonist). Recent studies indicate that endogenous molecules released from stressed or dead cells such as heat shock proteins (HSP; TLR2 and TLR4) and high mobility group box 1 (HMGB1; TLR2 and TLR4) are also important TLR agonists (Asea et al., 2002; Kepp et al., 2011).

When TLR agonists bind to their receptors, the bulk of downstream signaling is generally executed through one of two different pathways, myeloid differentiation factor 88 (MyD88)-dependent and MyD88-independent. The former leads to early activation of NF-κB, MAPK, and transcription of pro-inflammatory cytokines, chemokines, and cytosolic enzymes, while the latter results in the activation of late phase NF-κB and the interferon (IFN) regulatory factors responsible for type I IFN expression (Akira and Takeda, 2004; O’Neill and Bowie, 2007).

## Expression Of Tlrs In Microglia And Glioma Cells

For TLR agonist-based cancer immunotherapy, it is important to carefully evaluate the role of TLR agonist in terms of both systemic and regional effects in order to deliver the most effective treatment with the least number of side effects. Furthermore, while TLR expression on the immune cells generally supports the therapeutic purpose, the expression on cancer cells (Arunkumar et al., 2013; Cherfils-Vicini et al., 2010; Huang et al., 2007; Kundu et al., 2008; Nomi et al., 2010; Salaun et al., 2006) can corrupt the process. TLRs are expressed on both the tumor cells and microglia, a normal glial cell that makes up a major cellular component of human glioma (da Fonseca and Badie, 2013). The following section summarizes TLR expression patterns and related biological responses in order to predict potential regional effects elicited by TLR agonists in TLR agonist-based glioma immunotherapy.

### TLR expression on glioma cells

TLR2, TLR4, and TLR9 have been under investigation for expression on glioma cells, and their contribution to tumor development has been mostly described as tumor promoting.

#### TLR2

The expression levels of *TLR2* are substantially elevated in patient glioma biopsies and inversely correlate with patient survival (Vinnakota et al., 2013). *TLR2* expression has also been detected in human glioblastoma U87 cells with touchdown PCR (Haghparast et al., 2011) and subsequently linked to tumor promotion. When cells were treated with the TLR2 agonist peptidoglican (PGN), TLR2 initiated signaling through the activation of NF-κB, which ultimately led to increased cell growth (Echigo et al., 2012).

#### TLR4

*TLR4* RNA and/or protein expression has been detected in U118, U87, A172, and LN229 glioma cell lines (Gupta et al., 2013; Sarrazy et al., 2011; Tewari et al., 2012). Protein expression in primary biopsies from glioblastoma patients has been found to be elevated relative to adjacent non-neoplastic tissue by immunohistochemistry and Western blot analysis (Tewari et al., 2012). In *in vitro* studies, different tumor promoting effects were directly observed when cells were exposed to the TLR4 agonist LPS. First, the proliferation of U118 and U87 and the invasion of U87 cells (Sarrazy et al., 2011) were enhanced. The expression of the metalloproteinase, MMP-9, which is essential for increased invasion of the cells, was also elevated in U87 in response to stimulation by LPS. Second, signaling through TLR4 was found to be involved in mechanisms regulating cell survival, migration and immune evasion, and resistance to TNF-α treatment. TLR4 was either directly involved in these processes or influenced their outcome.

Enhanced invasion mediated by TLR4 is potentially the result of an intriguing mechanism that involves the binding of extracellular heat shock protein 90 (HSP90) to TLR4 with subsequent transactivation of epidermal growth factor receptor (EGFR). HSP90 is a major protein produced in response to tumor cell stress and is found at increased extracellular levels in the tumor environment. In this mechanism, HSP90 binds to TLR4, which leads to activation of EGFR and an increase in intracellular calcium levels necessary to promote tumor cell migration (Thuringer et al., 2011).

The release of specific cytokines that protect cells from apoptosis or lead to the ability of tumor cells to altogether evade immune detection may also be due to the expression of TLR4 on glioma cells. The resistance of gliomas to apoptosis, induced in one scenario by TNF-α, is possibly due to the release of cytokines IL-6, IL-8, and MCP-1, in fact, in response to TNF-α treatment (Tewari et al., 2009). IL-8 is associated with hypoxia induced glioma progression (Brat et al., 2005), and MCP-1 is responsible for the tumor promoting activity of microglia (Platten et al., 2003). In tumor cells, it is TLR4 that is activated by TNF-α, via TIR-domain-containing adapter-inducing interferon-β (TRIF) dependent pathway, forming a feed-forward loop with TNF-α, which triggers the release of these cytokines (Tewari et al., 2012). IL-1β is an additional cytokine secreted by glioma cells subsequent to TNF-α treatment. This cytokine actually induces TLR4 expression, via hypoxia inducible factor 1-α (HIF-1α), which results in the elevation of HMGB1 in glioma cells. The upregulation of TLR4 and HMGB1 is synergistic and results in the increase of HLA-G, a non-classical HLA class I antigen that participates in glioma immune evasion (Gupta et al., 2013).

One potential way to bypass these particular challenges due to the expression of TLR4 on glioma cells is through the activation of the Fas pathway. Engagement of this pathway generally leads to apoptosis in a variety of cancers (Shinohara et al., 2000), including glioma (Zhao et al., 2007). Interestingly, when TLR4 and Fas were simultaneously activated, the tumor promoting capabilities of TLR4 were lost, as well as the increased expression of MMP-9, which was necessary for the U87 cell invasion stimulated by LPS alone. The molecular details of how these two molecules/pathways functionally intersect requires further investigation, however, to be of therapeutic value (Sarrazy et al., 2011).

#### TLR9

TLR9 is expressed on human cell lines U251, U87, and the murine cell line C6. This receptor is also found in primary human glioma biopsies and isolated human glioma stem-like cells. Clinically, increased expression of TLR9 has been associated with higher tumor grade and worse prognosis. Functionally, studies have demonstrated that TLR9 mediates the properties of enhanced invasion and proliferation. Studies have demonstrated that the TLR9 agonist, CpG dinucleotide, enhances cell invasion *in vitro* through TLR9 (Wang et al., 2010). In addition, there is a correlation between TLR9 and the metalloproteinases MMP2 and MMP9 in clinical samples (Leng et al., 2012; Wang et al., 2010). Activated TLR9 also promoted growth of glioma stem-like cells through the activation of STAT3 signaling *in vitro*, which is a pathway participating in numerous tumor promoting activities (Herrmann et al., 2014). In contrast, CpG dinucleotides also function as a radio-sensitizer potentially through TLR9. CpG raises nitrogen monoxide (NO) levels in CpG treated U87 cells through TLR9 mediated NF-κB signaling, thereby potentiating the radiosensitivity of the cells *in vitro* (Li et al., 2012).

How TLRs might be controlled by other events occurring during tumor development is illustrated by the regulation of TLR9 by insulin growth factor 1 (IGF1). IGF-1 is a well-known growth factor associated with tumor growth. In glioma cells, IGF-1 induced TLR9 through HIF-1α signaling, mediated by Ras and calmodulin dependent kinase II (CaMK II). Enhanced expression of TLR9 induced by IGF-1 resulted in the secretion of IL-1β, IL-6, IL-8, and the elevation of CXCR-4, a receptor that plays an important role in cell migration. CXCR-4 may thus underlie the mechanism for TLR9 enhancement of the metastatic potential of glioma cells (Sinha et al., 2011).

In summary, TLR agonists bring potential risks to immunotherapies because these receptors can also initiate tumor promoting biological processes when expressed by glioma cells. For this reason, thorough studies of TLR expression in glioma cells, the effect of different TLR agonists for the same TLR or different TLRs on glioma cells *in vitro*, and the underlying molecular mechanisms are critical for the proper guidance and design of therapies.

### TLRs and microglia

Microglia is a type of tissue resident macrophage of myeloid origin and perform brain specific immune surveillance. These glial cells heavily infiltrate gliomas, and can constitute up to 30% of the infiltrating normal cell population (the stroma). The local density of microglia can be as great as 20 times higher in glioma than normal brain tissue (Hussain et al., 2006b). TLR2, 3, 4, and 9 are highly expressed on both human microglia in the normal brain parenchyma and tumor-infiltrating microglia. However, despite similar levels in TLR expression (Hussain et al., 2006b; Meng et al., 2008), the function of the infiltrating microglia is highly influenced by the tumor microenvironment.

TLRs play an important role in the regulation of microglial activity during gliomagenesis. For example, membrane type 1 matrix metalloproteinase (MT1-MMP) is a molecule in microglia that controls response to certain growth factors in neovascularization, and engagement of this molecule is one way that microglia contributes positively to glioma growth. Expression of TLR2 in microglia is necessary for the activation of MT1-MMP by neighboring glioma cells, and the process is MyD88 dependent (Vinnakota et al., 2013). Based on these results and the fact that the molecule is highly expressed in human gliomas, TLR2 was examined as a potential biomarker, and its expression was found to be inversely correlated to patient survival (Vinnakota et al., 2013).

Microglia isolated from patient biopsies, however do retain their antitumor properties. Poly(I:C) stimulation polarized isolated microglia *in vitro* toward an antitumor M1-like profile which resulted in increased microglial production of inflammatory cytokines TNF-α and IL-12 (Kees et al., 2012). These supernatants induced apoptosis and inhibited migration of glioma cells. The experiment was also performed in the presence of glioma cells to determine how the tumor microenvironment influenced microglial function. When co-cultured with glioma spheroids, poly(I:C) stimulated microglia were impaired with respect to their antitumor properties (Kees et al., 2012). Similar effects were observed when microglia was stimulated with a second TLR agonist, LPS. LPS also stimulated microglia to produce TNF-α *in vitro*, but glioma cells both through paracrine and direct cell-contact mechanisms, inhibited this effect. The study also revealed that the glioma cells induced microglia to secrete the anti-inflammatory cytokine IL-10 and to up-regulate STAT3 signaling activity, two activities that are tumor promoting (Kostianovsky et al., 2008).

TLRs mediate both tumor promoting and tumor inhibiting functions of microglia. Glioma cells skillfully usurp these glial cells for their growth promoting properties, and yet, microglia remains fundamentally unaltered with regard to their tumor inhibiting functions. Thus, microglia and TLR signaling are tools that may be manipulated for therapeutic purposes, particularly in light of the fact that these cells constitute a considerable fraction of human gliomas. Although engagement of TLR3 and TLR4 pathways alone, for example, may not be sufficient to eradicate tumors, targeting these receptors could be considered as a supplement to therapy until mechanisms of glioma cell suppression on microglia are more clearly understood.

## TLRS AND ANTI-GLIOMA IMMUNITY

Local immune suppression is one part of the challenges that face immunotherapy; immune suppression is a systemic manifestation of cancer as well. Research efforts to boost the immune response in cancer patients are therefore also focused on more fundamental failures of the immune system in cancers. Here, TLR agonists in glioma immunotherapies have also been promising as tools to activate APCs and effector T cells, and in general to reverse glioma induced suppression of maturation of these cell types. The following content reviews recent advances that describe how TLRs and TLR agonists contribute to the development of anti-glioma immunity.

### TLRs and glioma antigen presentation

DCs in the brain develop from pre-DCs located in meninges and choroid plexus (Anandasabapathy et al., 2011). Some investigators believe that DCs actually carry antigens from the CNS and migrate deep into the cervical lymph nodes (CLN) in order to activate the maturation of residing naive T cells (Karman et al., 2004). However, whether DC cells do migrate out of the CNS and how they do so, still remains controversial (Weller et al., 2009). Generally, DCs are thought to be the APCs fundamental to the development of effective anti-glioma immunity. Therefore, the role of TLR agonists in the maturation of DCs and the enhancement of their capacity for migration and antigen presentation is currently under rigorous investigation.

#### TLRs and DC activation

In contrast to its role in glioma-derived microglia, TLR2 expression on DCs is of potential benefit to immunogenic glioma cell lysis. Stimulation of the receptor by a corresponding endogenous or synthetic agonist has been shown to effectively initiate activation of DCs through NF-κB signaling. A novel method of locally producing a desired endogenous agonist was through gancyclovir mediated cell death of glioma cells expressing thymidine kinase from adenovirus. The release of HMGBI, a TLR2 agonist, from the dead glioma cells stimulated TLR2 (Foldi, 1999), and in combination with the local expression of the DC growth factor Fms-like tyrosine kinase 3 ligand (Flt3L) (Maraskovsky et al., 1996; Pulendran et al., 1997), led to expansion and activation of DCs resulting in the production of inflammatory cytokines TNF-α and IL-6 (Curtin et al., 2009) necessary to initiate an effective anti-glioma immune response (Ali et al., 2004). Weighing the balance in the dual character of TLR2 therefore appears to be an important consideration in the development of anti-glioma immunotherapies.

Systemic introduction of synthetic TLR agonists poly(I:C), imiquimod, and CpG also facilitated glioma antigen presentation. In an intracranial glioma model, imiquimod increased the number of DCs in the blood and CLN, and led to the doubling of the number of DCs in the brain (Xiong and Ohlfest, 2011). CpG was reported to activate and mature DCs as well (Krieg, 2006). Furthermore, a study in the murine model indicated that CpG achieved higher efficacy in the maturation of DCs when it was administered subcutaneously with tumor lysates. The combination exerted a synergistic effect in increasing the number of activated DCs in the cervical draining lymph nodes and eradicated intracerebral GL261 glioma cells in the mice (Wu et al., 2007). In a clinical trial, a DC vaccine with imiquimod or poly(I:C) as an adjuvant, boosted serum TNF-α and IL-6 levels in glioblastoma patients (Prins et al., 2011).

#### TLRs and glioma induced DC suppression

The greatest challenge to immunotherapy is to overcome the local immunosuppressive microenvironment. TGF-β secreted by tumor cells is one of the most important and extensively studied factors contributing to glioma microenvironment immune suppression (Kjellman et al., 2000). TGF-β enables the tumor to efficiently evade immune surveillance by potently suppressing the maturation and function of DCs, which leads to a deficiency in antigen presentation and immune response (Grauer et al., 2007). Any effective immunotherapy, thus, must enable DCs to both mature and produce IL-12 in order to produce cytotoxic/mature T cells. TLR7 agonists that directly target DCs have been used in an attempt to reverse this immune suppression. R848 (TLR7/TLR8 agonist) in a cocktail with TNF-α, IL-1β, and IFN-γ induced DC maturation and the production of IL-12 (Grauer et al., 2007).

Factors in addition to TGF-β are also likely to contribute to the immune suppressive milieu in glioma, and through novel mechanisms. In one study, DCs isolated from fresh glioma biopsies were found to be refractory even to TLR agonist stimulation *in vitro* (Hussain et al., 2006a). A separate study identified lactate as an additional molecule that suppresses the immune response, and thus is also responsible for the resistance of DCs to TLR agonist stimulation (Chirasani et al., 2013). Lactate is abundant in glioma and most aggressively proliferating tumors (Walenta and Mueller-Klieser, 2004). Lactate decreased IL-12 production from mouse intracranial glioma derived DCs stimulated by R848, and even altered the function of the agonist, which resulted in the activation of STAT-3 signaling in DCs and subsequent suppression of their maturation. Small molecule inhibition of lactate enabled the TLR7/8 agonist R848 to stimulate secretion of IL-12 by DC in the glioma environment thereby alleviating immune suppression (Chirasani et al., 2013).

### TLRs and anti-glioma immune response

After successful antigen presentation by DCs, effector T cells are activated to kill glioma cells. Their levels are subsequently redistributed in the tumor, brain, CLNs, and blood. TLR agonists delivered either systemically or locally, with or without tumor lysate/antigens, have achieved observable changes in the levels of these immune cell types in a variety of preclinical murine glioma models, that resulted in either eradication of the tumor or suppression of tumor growth. The main players involved in anti-glioma TLR immunotherapy are CD8^+^ T cells, which are assisted by CD4^+^ T cells. In certain studies however, the effector cells are not limited to the adaptive immunity. Members of the innate immune system, such as NK cells, also make important contributions depending on the agonist used and the therapeutic approach chosen (Alizadeh et al., 2010; Zhao et al., 2011). Cytokine changes can be associated with tumor suppression and under certain conditions, present independent factors that must be taken into consideration. Positive immunotherapeutic roles elicited through a variety of mechanisms have involved TLR2, TLR3, TLR4, TLR7, and TLR9 in these cell types.

#### TLR2

Bacterial lipoprotein (TLR2 agonist) administered with antigen-specific T cells achieved long-term survival and immune memory in the murine GL261 glioma model (Zhang et al., 2014). In this study, a TLR1/TLR2 agonist enhanced the survival and function of administered T cells and altered the glioma microenvironment by simultaneously elevating the number of CD8^+^ positive T cells and down-regulating the number of immunosuppressive myeloid-derived suppressor cells (MDSCs). This effect was TLR2 dependent. In other experiments, endogenous TLR2 agonist HMGB1, released from glioma cells by targeted treatment with gancyclovir, activated DCs and facilitated therapy (Curtin et al., 2009). Under this treatment strategy, nearly half of the mice bearing intracranial GL261 gliomas achieved long-term survival and anti-glioma immunological memory in a TLR2 dependent manner. The therapy stimulated the clonal expansion of glioma antigen specific T cells in tumor bearing mice, with a significantly higher proportion of T cell precursors releasing IFNγ in response to glioma antigen.

#### TLR3

In the murine CNS model, poly(I: C) enhanced induction, expansion, and CNS homing of antigen specific T cells. CD8^+^ positive T cells in CLNs displayed higher expression of alpha4 beta1 integrin (very late activation antigen 4, VLA4), a molecule that favors CNS tropism (Calzascia et al., 2005). Poly(I:C) facilitated tumor antigen specific T cell homing via VLA4 up-regulation. In addition, poly(I:C) stimulated T cells to produce more IFN-γ in the intracranial glioma microenvironment (Zhu et al., 2007).

#### TLR4

Two TLR4 agonists, LPS and Spirulina complex polysaccharide, have demonstrated efficacy in mice with subcutaneous implantation of DBT and RSV-M gliomas, respectively (Chicoine et al., 2007; Kawanishi et al., 2013). TLR4 signaling was activated and a variety of immune cell types, including CD4^+^ T, CD8^+^ T, NK cells, and macrophages, were exploited in tumor suppression. One major difference between the mechanisms of glioma suppression utilized by LPS and Spirulina complex polysaccharide in glioma suppression is that LPS elevates IL-17 in mouse serum while Spirulina complex polysaccharid downregulates the cytokine. IL-17 is a pleiotropic cytokine with pro- or anti-tumor potential depending on the tumor model. In the murine glioma model, IL-17 was shown to promote glioma growth (Chicoine et al., 2007). Moreover, Spirulina complex polysaccharide was less toxic. These particular studies on TLR4 agonists also highlight the importance in general of optimizing their utility, such as lowering toxicity while maintaining antitumor activity, in a clinical setting.

#### TLR7

Topical administration of the TLR7 agonist imiquimod has been approved by the FDA in the treatment of skin cancers and genital warts. In one of the more unusual treatment approaches, topical administration of imiquimod (shoulder and flank) was also employed in a mouse glioma model and in fact eradicated the intracranial tumor (Xiong and Ohlfest, 2011). Topical administration in the mice depleted CD4^+^ T and CD8^+^ T cells in the peripheral blood circulation, redistributed them in mouse CLNs and brain, and reversed tumor immune suppression by significantly reducing the absolute number of Tregs in the tumor microenvironment. These responses all together led to intracranial tumor inhibition. The topical administration of imiquimod also released/activated brain infiltrating lymphocytes from the refractory state induced by the intracranial tumor (Xiong and Ohlfest, 2011). Moreover, antitumor immune memory developed in mice subcutaneously injected with imiquimod (Stathopoulos et al., 2012).

#### TLR9

The TLR9 agonist CpG has been shown to eradicate murine intracranial glioma when subcutaneously administered with tumor lysate and effector T cells (Wu et al., 2007). Further investigation into local administration of CpG analyzed the change in the balance between effector T cells and regulatory T cells at the tumor site. The ratio of CD8^+^ T effector cells to CD4^+^FoxP3^+^ Tregs in the tumor environment was shown to be increased. In CLNs, antigen-specific activation of CD4^+^ and CD8^+^ T cells was observed with CpG administration (Grauer et al., 2007).

Side effects, such as a transient worsening of baseline neurological symptoms resulting from brain edema (Calzascia et al., 2005; da Fonseca and Badie, 2013), occurred in the CpG clinical trial. One clinical study attempted to avoid the severe neural inflammation caused by the single high dose injection by applying CpG through multiple injections at a lower dose. In this study, NK cells were found to be increased and were higher than CD8^+^ T cells in the tumor. Depletion of NK cells with antibody completely abrogated the antitumor effect, while only a minor reduction in efficacy was observed after CD8^+^ T cell depletion (Alizadeh et al., 2010). Another important finding of this study was the significant elevation of MDSCs, but not Tregs, in the brain, blood, and spleens in response to the single high dose injection of CpG-ODN. This finding supports the administration of multiple low dose injections of CpG rather than a single high dose injection (Alizadeh et al., 2010). Other methods of drug delivery, carbon nanotube application, appeared to also enhance the efficacy of CpG, including a survival benefit. The study also confirmed the roles of CD8^+^ T cells and NK cells in the anti-glioma immune response (Zhao et al., 2011).

Based on the current literature, TLR3 and TLR7 agonists may be generally categorized as “safe” agonists to use in glioma therapy, as response is largely tumor inhibition in the absence of tumor promotion. However, evidence of TLR3 and TLR7 tumor-promotion has been reported for other cancers such as in lung, pancreas, and liver (Chatterjee et al., 2014; Eigenbrod and Dalpke, 2013; Eiro et al., 2014; Zhan et al., 2014). Further investigation is, therefore, necessary to confirm these observations. In the case of TLR4 and TLR9 agonists, the recommendation is to remain cautious when considering their roles in anti-glioma therapies. Results already demonstrate that *in vitro* and *in vivo* studies do not completely overlap. One important line of investigation for the future is to perform *in vivo* studies of human glioma cell lines in humanized mouse models in order to fully understand the nature of the anti-tumor and pro-tumor effects of TLR4 and TLR9 agonists.

## CLINICAL TRIALS BASED ON TLR AGONIST THERAPY

### Biological rationale for immunotherapy in the central nervous system

The human central nervous system was once regarded as immunologically privileged. Several factors that constitute an effective immune system were thought to be absent or insufficient in the CNS. They included the mode of entry for immune cells which was thought to be blocked by the blood brain barrier (BBB), the lymphatic vascular system, and major histocompatibility complex (MHC) and local antigen presenting cells (Muldoon et al., 2013). Current understanding has replaced the previous concept of the CNS immune system, as a result of the discovery of some CNS specific mechanisms for eliciting immune response. First, the BBB is compromised in pathological conditions such as inflammation and tumor development, and under pharmacological modification with BBB specific disruptive agents (Bartus et al., 1996; Boskovitz et al., 2004; Elliott et al., 1996; Stamatovic et al., 2008; Wang and Casley-Smith, 1989; Wolburg et al., 2005). Any one of these conditions thus theoretially provides the opportunity for entry of both immune cells and therapeutic monoclonal antibodies. Second, instead of lymphatic drainage in the traditional sense, CNS immunological contact with its draining CLN is established through the perineural lymphatic pathway (Foldi, 1999) and the prelymphatic-lymphatic system (Wang and Casley-Smith, 1989). Third, CNS residing microglia, with a poor antigen presentation ability in the resting state, could be stimulated by an underlying pathology in the CNS to interact with T cells and become effective APCs (Yang et al., 2010). In addition, macrophages and especially DCs, though limited in the brain parenchyma, are abundant in choroid plexus epithelium and serve as strong APCs under certain biological and pathological conditions (Hussain and Heimberger, 2005; Serot et al., 1997). Finally, the CNS has its own immune surveillance system. The immune cells enter the cerebrospinal fluid (CSF) at the position in the choroid plexus where CSF is produced, and CSF circulation establishes immune surveillance (Provencio et al., 2005).

### Current status of clinical trials based on TLR agonist therapy

An important aspect of the utilization of TLRs in glioma clinical trials is the application of TLR agonists as single agents to suppress the tumor. A number of trials have been designed based on this strategy, and some have supplemented TLR treatment with chemo- and radio-therapies. To date, three clinical trials have been completed with published results.

One CpG phase II clinical trial has been completed for patients with recurrent GBM. A total of 34 patients with recurrent GBM were enrolled; CpG was administered intracerebrally to 31/34 patients. Overall, the therapy was well tolerated. The 6-month progression free-survival (19%) was increased compared to the control group (3%). One partial response (−78%) and 3 minor responses (−33%, −26%, and −25%) were observed. Adverse events associated with therapy included lymphopenia, mild fever, seizures, and worsening of baseline neurological symptoms (Carpentier et al., 2010).

Two Phase II polyICLC (a chemically and biologically stable form of poly(I:C)) trials have been completed. In one clinical trial, which evaluated the therapeutic efficacy of polyICLC in combination with irradiation for newly diagnosed GBM patients, muscle injection of polyICLC was administered to 30 patients. The treatment achieved a survival benefit compared to historical studies using irradiation without chemotherapy, but no survival benefit compared to irradiation with adjuvant nitrosourea or non-temozolomide chemotherapy. Toxicity was generally minor, but could be accompanied by fatigue, leucopenia, lymphocytopenia, and pain at the site of injection (Butowski et al., 2009). PolyICLC was further tested as adjuvant therapy on postoperative glioblastoma patients in combination with the current standard for treatment, irradiation and temozolomide (TMZ). At the endpoint of the trial, the overall survival at 12 and 18 months was greater than in the previous EORTC phase III trial in which patients were treated with irradiation and TMZ (Rosenfeld et al., 2010; Stupp et al., 2005).

In many other clinical trials, TLR agonists are designed to be administered with other glioma vaccines as adjuvant. Most of these clinical trials focus on tumor antigens and DCs (Jackson et al., 2013). Several antigens or glioma specific molecules have been characterized and utilized in glioma vaccines, including epidermal growth factor receptor variant III (EGFRvIII), IL-13 receptor α2, IL-4 receptor, and heat shock protein (HSP) gp96 (Eguchi et al., 2006; Graner et al., 2007; Kawakami et al., 2000; Li et al., 2010). However, instead of single antigens, many more designs utilize a mixture of antigens, namely tumor lysate that contains most of the glioma antigens that have been discovered. Furthermore, regarding DCs in vaccines, the most important APCs are artificially derived in an *in vitro* environment to enhance contact between the cells and the antigens. TLR agonists are thereby designed into the procedure to augment the therapeutic effect.

Table 1 lists all current clinical trials for TLR agonists in glioma immunotherapy. Most of these trials are still at the patient recruiting stage. The trials are mainly focused on the therapeutic utility of agonists for TLR3, TLR7, and TLR9. The targets of the immunotherapy include primary and recurrent gliomas of different grades and pathology from pediatric as well as adult patients. Identifier numbers for the clinical trial are listed in the table, and for more detailed information, please refer to the government website: http://www.clinicaltrials.gov.

**Table 1:** Clinical trials in glioma immunotherapy with TLR agonists

Diagnosis	TLR target	Agent	Phase	Status		Trial
GBM	TLR3	PolyICLC/ APVAC1 vaccine	1	Not recruiting		NCT02149225
AIII, GBM	TLR3	PolyICLC/IMA950	1/2	Recruiting		NCT01920191
Recurrent Ped LGG	TLR3	PolyICLC	2	Recruiting		NCT01188096
Recurrent AII, OII, OAII	TLR3	PolyICLC/glioma peptide	0	Not recruiting		NCT00874861
Recurrent GBM, AIII, OIII, OAIII	TLR3	PolyICLC/DC vaccine	1/2	Not recruiting		NCT00766753
AIII, OAIII, GBM	TLR3/TLR7	PolyICLC/Resiquimod/DC vaccine	2	Recruiting		NCT01204684
Ped pontine glioma, ped HGG, recurrent ped LGG, recurrent ped HGG	TLR3	PolyICLC/ glioma peptide	–	Recruiting		NCT01130077
GBM/post operative	TLR3	PolyICLC/radiation	2	Completed		NCT00052715
AIII, OIII, OAIII	TLR3	PolyICLC	2	Unknown		NCT00058123
GBM/post operative	TLR3	PolyICLC/Temozolomide/radiation	2	Completed		NCT00262730
High risk AII, OAII	TLR7	Imiquimod/Tumor lysate vaccine	0	Recruiting		NCT01678352
Recurrent Ped ependymoma	TLR7	Imiquimod/glioma peptide	2	Recruiting		NCT01795313
Recurrent GBM, AIII	TLR7	Imiquimod/DC vaccine/Tumor lysate	2	Recruiting		NCT01808820
GBM	TLR7	Imiquimod/SL-701 vaccine/Leukine 150 micrograms	1/2	Recruiting		NCT02078648
GBM, AIII, Medulloblastoma, Ependymoma	TLR7	Imiquimod/DC vaccine(tumor stem cell loaded)	1	Completed		NCT01171469
PedGBM, HGG	TLR7	Imiquimod/DC vaccine/Tumor lysate	1	Recruiting		NCT01902771
GBM	TLR9	CpG-ODN	2	Completed		NCT00190424

*A* astrocytoma, *O* oligodendroglioma, *OA* oligoastrocytoma, *GBM* glioblastoma multiforme, *ped* pediatric, *LGG* low grade glioma, *HGG* high grade glioma

Finally, new developments emerging from studies on TLR agonists in many other tumor models may also be relevant to immunotherapy for glioma patients. Firstly, tumor antigens for vaccines are derived from lysates of cancer stem cells in order to more aggressively generate an anti-tumor response. In a murine model of triple negative breast cancer, CpG was administered as an adjuvant in a vaccine based on a breast cancer stem cell lysate, which achieved tumor regression and a survival benefit (Liu et al., 2013). Secondly, combination therapies of different TLR agonists have been reported to exert synergistic stimulatory effects upon anti-tumor immune response. Co-delivery of 3 M-052 (TLR7, TLR8 agonist) and CpG ODN via intratumoral injection has led to complete rejection of large tumors (colon cancer and melanoma) in murine models, while delivery of 3 M-052 or CpG ODN alone only reduced the tumor growth rate (Zhao et al., 2014). Thirdly, TLR agonists have been combined with therapeutic agents targeting certain immunosuppressive factors. BCG administered with IL-10 receptor 1 monoclonal antibody resulted in enhanced anti-tumor immunity in a murine model of metastatic bladder cancer, which had been initially resistant to BCG treatment alone (Newton et al., 2014). Similar findings were reported from a study on a murine lymphoma model treated with CpG and IL-10 siRNA (Pradhan et al., 2014). Lastly, efforts are being made to enhance anti-tumor response with improvements in the delivery system for TLR agonists, such as with cationic liposomes. Cationic liposomes have been found to potentiate anti-tumor immune responses induced by poly(I:C) in murine melanoma models (Hansen et al., 2012), and to engage TLR4 on tumor associated macrophages that also resulted in anti-tumor immune response (Huang et al., 2013). A more novel approach for delivery exploited a type of nanogel which was loaded with CpG ODN or polyICLC and peptide antigen. This combination was reported to be capable of specifically delivering the vaccines to the draining lymph nodes and preferentially activating the medullary macrophages (Muraoka et al., 2014), which resulted in effective cross-priming of vaccine specific effector T cells and anti-tumor activities in murine colon cancer and sarcoma models.

## CONCLUSIONS

**Figure 1:**
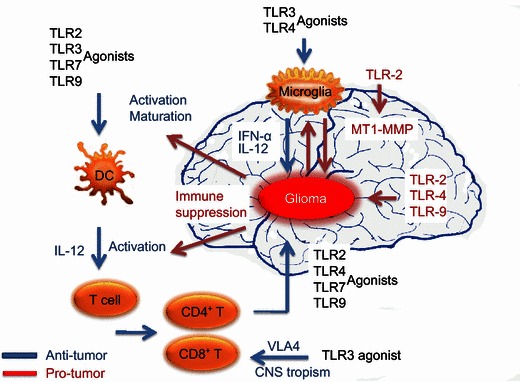
**TLR agonists stimulate both anti- and pro-glioma processes**. Biological activities initiated through TLR pathways are dependent on the receptor stimulated, the cell type, and the microenvironment. In the anti-glioma process, TLR agonists encourage the development of an immune environment by directly stimulating DC and T cell maturation. In addition, TLR activation in these cell types opposes the immune suppressive environment by favoring CNS tropism. Migration elevates the numbers of these cells in the CNS tumor and CLN and initiates anti-glioma immunity. TLR agonists also induce microglia isolated from primary gliomas to produce inflammatory cytokines and acquire anti-glioma activity. In contrast, activation of TLR2, TLR4, and TLR9 in microglia facilitates pro-glioma activities, such as tumor cell growth, invasion, and migration

Immunotherapy designed against human gliomas is fundamentally challenged by the sophisticated interactions between gliomas and their immunological environments. In addition, TLRs are distributed widely among cell types and can elicit completely different responses depending on the cell and the environment (Fig. 1). Most studies fractionate these differences by focusing on one TLR and one tumor cell type as the target for study. However, the nature of the widespread expression and the combined effect of TLRs and TLR agonists underlie the necessity for a more comprehensive understanding of their function in glioma therapies. Results from many biological disciplines have contributed to our current molecular and clinical understanding, but information is still lacking, and many questions await answers. What is the general distribution of glioma TLR expression on an epidemiological scale? What are the real clinical effects of TLR agonists on glioma patients? How do we select and administer TLR agonists and maximize their anti-tumor effect while minimizing their pro-tumor potential? Is it possible or necessary to adopt a combination(s) of different TLRs and agonists in glioma treatment? Answering these questions requires further molecular and clinical investigation, which will undoubtedly lead to new, perhaps groundbreaking insights for the clinical management of human glioma.
